# Work-life balance Twitter insights: A social media analysis before and after COVID-19 pandemic

**DOI:** 10.1016/j.heliyon.2024.e33388

**Published:** 2024-06-22

**Authors:** Kateřina Kuralová, Kristýna Zychová, Lucie Kvasničková Stanislavská, Lucie Pilařová, Ladislav Pilař

**Affiliations:** Department of Management and Marketing, Faculty of Economics and Management, Czech University of Life Sciences Prague, Czech Republic

**Keywords:** COVID-19 pandemic, Frequency analysis, Future of work, Hashtag research framework, Human resource management, Labour market, Sentiment analysis, Social media analysis, Topic analysis, Twitter, Work-life balance

## Abstract

This research examines the perceptions of Twitter users regarding the prevalent topics within Work-Life Balance communication before and after the COVID-19 pandemic. The pressing questions surrounding current labour market drivers are addressed, particularly regarding the ongoing Fourth Industrial Revolution and the COVID-19 pandemic's impact on communicated themes, particularly in the Human Resource Management field, where Work-Life Balance has emerged as a key concept. Social media platforms like Twitter are pivotal in fostering discussions on Work-Life Balance in society. Over the past decade, Twitter has evolved into a significant research platform researchers utilise in more than ten thousand research articles. The online discourse on Twitter raises awareness of the importance of balancing work and personal life. The COVID-19 pandemic has unveiled new facets of Work-Life Balance, with social media as a key platform for discussing these issues. This research uses Social Media Analysis based on the Hashtag Research framework. A total of 1,768,628 tweets from 499,574 users were examined, and frequency, topic, and sentiment analysis were conducted. Pre-pandemic, the most communicated Work-Life Balance topics were performance and time management, while recruitment and employee development were identified post-pandemic. Pre-pandemic, the highest proportion of negative sentiment was time management and mental health prevention, shifting to time, employee development, and mental health prevention post-pandemic. Despite the limitations of our research, a proposed redefinition of the concept is also presented, including a design for an integrated Work-Life Balance model based on topics communicated by Twitter users. Given the need for a more robust approach to redefining the concept and developing an integrative Work-Life Balance model, the article provides fresh insights for future research.

## 1 Introduction

The evolving nature of employment and labour creates new societal demands. One of these demands is employee Work-Life Balance (WLB). As an essential aspect of employment relations, WLB contributes to a healthy work environment that significantly impacts employees and their families, organisations and society [1].

With the contribution of the Fourth Industrial Revolution (Industry 4.0), marked by digitalisation and robotic technologies, with an uncertain but potentially huge impact on employment, the global employment crisis is still ongoing, with no end. Technological progress may exacerbate a problematic situation in the short term [2], further intensified by the COVID-19 pandemic (COVID-19) [3,4] or international conflicts [5]. According to Vyas [6], the COVID-19 has significantly disrupted labour markets and triggered an immediate wave of various experiments with flexible work arrangements and new ways of moving towards a centralised working environment. As Vyas [6] pointed out, approaches to reshaping working hours have laid the foundation for the so-called ‘new normal’, which is spreading to the field of work organisation in the post-pandemic era. These new arrangements, particularly flexible working arrangements, have challenged traditional employee-employer relationships, working hours, number of hours worked and WLB, including the individual relationship to work. According to Guest [7], with the advancement of technology, demographic changes, international labour mobility and the blurring of geographical boundaries, WLB has gained a vital role in Human Resource Management (HRM). Similarly, Gillnerová et al. [8] assert that the issue of WLB has come to the fore mainly due to the change in demographic trends and the shortage of skilled labour. Smith and Gardner [9], drawing on Brough and Kelling [10], Frone et al. [11], Frone and Yardley [12] and Hobson et al. [13], argue that demographic changes, including an increase in the number of women in the workplace, two-career families, single-parent families, and an ageing population, are leading to an increasingly diverse workforce and a greater need for employees to balance their work and personal life. As Khallash and Kruse [14] pointed out, employees are in an age of transition, driven by technological opportunities and the feminisation of the workforce. Europe face challenges related to economic, social and demographic changes that will affect the future organisation of work and the concept of WLB [14].

Nowadays, for many employees, social media is a powerful communication tool that helps them to collaborate, share ideas and solve problems [15]. In the context of the burgeoning popularity of social media, which is anticipated to encompass 5.85 billion users by 2027 [16]. Panuš [17] lists specific areas in which social media plays an important role and states that there are many reasons for using social media in research. Consistently according to Himelboim [18], the study of social media networks has evolved over the last two decades into an interdisciplinary scholarly area of research. Network analysis of social media data emerged in the late twentieth century when massive social interactions were recorded for the first time in history and became accessible to researchers [18]. The pursuit of comprehending the essence of social media content is shared by researchers and governments and organisations seeking to fathom the developmental trends of social consensus through social media content analysis [19]. Social media data analysis has characteristics of big data that differ from the data analysis methods traditionally used by social scientists and previously applied by mass communication researchers [20]. As a result, social media data analysis is a current area of researchers’ interest and requires further attention [21].

Despite the increased attention of researchers to social network data analysis, no study has yet been published on the mass communication of WLB using Twitter content analysis. Existing studies that link WLB to the topic of social media have primarily focused on the impact of social media use on WLB and the well-being of its users [[22], [23], [24], [25], [26]]. Thus, social media analysis emerges as an invaluable tool for understanding the dynamics of WLB. As social media have become an integral part of daily life for a vast segment of the population [16], it enables the identification of attitudes, opinions, and values expressed by users concerning this topic. This accessibility to a wide range of user-generated content offers a unique perspective into the public discourse surrounding WLB, making social media a rich resource for qualitative and quantitative analysis in this field.

This research aims to use social media analysis to identify the hashtags, the most common words and the key topics that Twitter users communicated about WLB before and after the COVID-19, including sentiments evoked by discussions about the communicated topics. Thus, this research reveals the dynamics of Twitter users' communication of the WLB topic during the research period.

Further, we also provide a proposal for redefining the concept of WLB, including a design of an integrated WLB model in the context of HRM practice. By proposing a new definition and an integrated model of WLB, we summarise the current findings, open the discussion to the researcher community and encourage possible future research directions. The proposed redefinition and integrated model in the context of future work serve as a basis for future research and indicate a way forward in defining the concept of WLB. Methodologically, we offer a new perspective to the field of WLB research by providing an example of how big data and social media analysis can be used in this area. Thus, the research uses social media analysis in the context of the communication of WLB themes on Twitter before and after the COVID-19 with possible implications for the future of HRM in the workplace.

Our article brings several novel contributions that distinguish it from previous studies in the field. The first novelty is using of social media as a data source for WLB analysis. While there have been studies examining the impact of social media use on WLB and the well-being of its users, our work focuses on the mass communication of WLB on the social network Twitter using content analysis. This approach allows for identifying key themes and user attitudes on this issue, providing a unique perspective on the public debate on WLB. The second innovation is the proposed redefinition of the WLB concept and the design of an integrated model of WLB in the context of HRM practices. By attempting to define WLB in the new context of future work after the COVID-19 and by presenting an integrated model that incorporates key HRM practices that support WLB, we provide a new perspective on the issue and open the debate on this topic. Another novelty is the methodological approach. Our article presents a new perspective on WLB research by demonstrating the use of big data and social media analysis in this field. This method enables a deeper understanding of the dynamics of WLB communication on Twitter and provides an opportunity for further research in this area.

## 2 Theoretical background

According to Storm and Muhr [27], many individuals today lead busy lives and spend significant time at work, including remote work. This can make it difficult to separate work and personal life, resulting in challenges with WLB and increased stress. This instability can negatively impact work performance and adversely affect the organisation. As a result, organisations face the challenge of finding ways to help employees balance their personal and professional lives to prevent burnout and employee turnover [28,29].

In the present era of the knowledge society, a satisfied and motivated employee is essential for the successful functioning of organisations [30]. Therefore, it is crucial to integrate social policy tools into their overall functioning. People can be seen as an organisation's most valuable asset [31]. According to Mescher et al. [32], qualified and reliable employees are scarce in the labour market and difficult for employers to obtain. The ability to balance work and personal life becomes one of the critical decision criteria for these employees when choosing an organisation. WLB measures are often regarded as an employee benefit in an organisation. WLB practices are HRM activities that employers can use to attract or retain talented people [32]. According to Yu et al. [33], WLB is essential for all employees regardless of age, gender, education level, family structure or occupation.

According to Rožman et al. [30], employers’ attention towards WLB measures helps to maintain employee motivation and performance. However, employees must be disciplined and well-organised in managing their work and personal lives. Wolor et al. [34] pointed out that the global health crisis has made people pay more attention to health and mental hygiene, increasing the demand for healthy workplace culture. However, to achieve WLB for their employees in a post-COVID-19 world, employers will need to consider and plan strategies to communicate the importance of WLB towards employees, including developing specific WLB programmes [34].

### 2.1 Labour market trends and HRM

According to the International Labour Organization (ILO) [4], global recovery from the COVID-19 crisis remains incomplete and uneven. The recovery is further complicated by the repercussions of the conflict in Ukraine, the accelerating climate change, and unprecedented humanitarian challenges. Estimates of slowing economic and employment growth in 2023 suggest that most countries will not fully restore to pre-pandemic levels in the foreseeable future [4]. Lack of access to employment, poor quality of work, inadequate remuneration and significant inequalities are cited by ILO [4] as problems with adverse effects on social equilibrium. Emphasis is placed on global solidarity and improving policy coherence to promote decent work and social justice.

According to Bartram [35], the prominent trends affecting people management in 2023 will be new labour standards in flexible working and teleworking, introducing the four-day working week and financial strategies to cope with economic uncertainty. Bartram [35] also discusses the practices that HRM professionals must develop and mentions the importance of employee feedback to deal with these changes successfully. Chappuis [36] also discusses HRM professionals' role in shaping the future of work and mentions digitalisation trends, Artificial Intelligence (AI), Machine Learning (ML) and robotics. Chappuis [36] presents ten future features and key characteristics for HRM professionals to work effectively and suggests five specific recommendations. Da Silva et al. [37] look closer at the digitalisation trend, which they believe is changing the work environment and HRM. Introducing disruptive technologies associated with the Industry 4.0 which changes how people work, learn, lead, recruit, and interact [37]. The digital trends arising from it, impact the HRM sector on many topics and support trends and challenges for HRM, employees and organisations. Da Silva et al. [37] presented a theoretical framework and identified different macro-groups related to HRM 4.0 (HRM in the context of Industry 4.0): HR-Digitalisation, HR-Management, HR-Strategy and HR-Competence. Trends arising from these groups include trends concerning the strategic value of people, digital technologies that drive HRM (AI, ML, Virtual Reality (VR) and big data), trends in lifelong learning and the inclusion of autistic and older people, changes in the image of the organisation to retain talent, new adaptations in the workplace, digital leadership, adaptive culture and new competencies for Workforce 4.0. Patel [38] further addresses this topic of workplace trends. According to Björk-Fant et al. [39], even though it has long been recognised that the socio-economic context shapes work stress and work-related mental health problems, and it is therefore considered a public health issue in Europe, their results suggest that this is also true for work well-being and related supporting factors. Ammirato et al. [40] examined the structure of the existing literature on Industry 4.0 and HRM in terms of major research themes and identified three main topics of current research interest that also represent areas for future research. (1) ‘*Digitalisation perspective: Industry 4.0 technologies for* *HRM* *and Innovation*’ which concerns how Industry 4.0 technologies support 10.13039/501100014832HR managers to design and monitor the tasks, working conditions, and activities of the workforce, (2) ‘HRM *4.0 − management and organization perspective: improving performance with Industry 4.0 technologies*’ which concerns the potential of Industry 4.0 technologies to improve workforce and HRM performance throughout the different phases of the 10.13039/501100014832HR lifecycle and (3) ‘HRM *5.0 − Criticisms of Industry 4.0 and discussions regarding Industry 5.0 considering sustainability challenges*’ which concerns the strategic implications of implementing Industry 4.0 technologies from a human-centric and sustainability perspective.

The ILO [41] states that the number of hours worked, how they are organised, and the availability of rest time can significantly affect the quality of work and life outside the workplace. Working hours and the organisation of working and rest periods can substantially impact employees’ physical and mental health and well-being and their safety at work and when commuting from home to the workplace [42]. Working time also has important implications for organisations regarding their performance, productivity and competitiveness [43].

Further, according to ILO [43], decisions on working time issues can also impact the economy's broader health, industry competitiveness, employment and unemployment levels, the need for transport and other facilities and the organisation of public services. Working time through measures such as part-time/work-sharing arrangements and flexible working are vital tools that can counter the threats posed by economic crises. At the same time, teleworking can reduce the social and economic impact of pandemics such as the COVID-19. It is not surprising that working time issues are at the heart of most of the labour market reforms and developments in the world today in one form or another [41]. According to Eurofound [44], ensuring an appropriate WLB is important not only for employees' health and well-being but also for increasing women's participation in the labour market. Eurofound researches the impact of Information and Communication Technologies (ICTs) and telework and ICTs-based mobile work on working conditions [45,46] and WLB [47,48]. Eurofound [44] considers the development of ICTs as the primary driver of changes in working life over the last two decades, with developments in ICTs contributing to new methods of work organisation by providing greater flexibility in the performance of work tasks. There is a shift in working life from regular, bureaucratic and ‘factory’ working time patterns to more flexible ones. Eurofound [44] states that teleworking and mobile working based on ICTs exemplify how digital technologies have led to more flexible working conditions and working time patterns.

### 2.2 WLB definitions

Adisa et al. [49] cite Kanter [50] who, over 40 years ago, identified the so-called ‘myth of separate worlds’ and states that he drew attention to the fact that work and non-work (especially home/family) domains are inextricably linked. Clark [51] discusses the broader theory, in which the boundaries between the work and non-work disciplines are critical for creating and maintaining a satisfying balance.

One immediately apparent issue with current WLB research is the lack of a standardised definition for the term, with many researchers approaching the phrase with varying conceptualisations and no set benchmark understanding from which to operate [[52], [53], [54]].

As Dhas [55] explains: ‘*Researchers need to recognise that balance can have both an objective and subjective meaning and measurement, that it will vary according to the circumstances, and that it will also vary across individuals*’. Different employees will view various factors as disruptive to their perceptions of their WLB, with such factors including conflict between work needs and family needs (including caregiver strain related to providing care for children or elderly parents), the amount of time spent at work versus at home, role overload, job security, support or lack of support from a supervisor or colleagues, ambiguity in their work role, job satisfaction, and the increasing use of various forms of communication technology, which can lead to an expectation that work tasks can be completed regardless of whether an employee is physically at work or ‘on the clock’ [55]. Kalliath and Brough [56] proposed a definition of WLB that an employee experiences a sense of WLB when he or she possesses an ‘*individual perception that work and non-work activities are compatible and promote growth in accordance with an individual's current life priorities*'. In contrast, Le et al. [57], drawing on prior research from Hill et al. [58], proposed an entirely new term for WLB: work-life interface (WLI). This term is described as ‘*a catch-all term for work-life (or family, home) balance, conflict, interaction, interference, enrichment, facilitation, and spillover. The WLI is conceptualised to include both negative (*e.g.*, work-family and family-work conflict) and positive (*e.g.*, work-family and family-work enrichment) foci*’ [57].

The lack of a standardised definition for the term is not the only issue confronting the conceptualisation of WLB in the academic literature. Despite the surging popularity of the use and discussion of WLB concepts and strategies within various industries and societies, intellectual discourse and theoretical development of the concept itself have not progressed at the same pace [53,59,60]. WLB has also primarily been analysed through a Western lens, meaning that current definitions of WLB are often informed purely by a Western context and do not apply to work cultures outside the West, such as within Asia [57]. In the Chinese context specifically, the importance Chinese society places on guanxi (defined by Chua et al. [61] as personalised social networks of power that overlap between different domains in an individual's life) leads to a merging of work and non-work life activities in China that is not only normalised, but expected for an employee to be recruited, retained, and promoted in many Chinese industries [57,62,63]. Additionally, work cultures in many Asian countries result in employees working longer hours and possessing fewer work holidays than employees in the West,and they lack many legal protections for employees' rights, which are considered a given in many Western societies [[64], [65], [66]]. Therefore, approaching the definition of WLB as though the terminology represents the same lifestyle and working arrangements across different cultures and societies means ignoring the nuances of varying work expectations within various countries.

### 2.3 WLB initiatives and benefits

There is generally no single accepted definition of what constitutes WLB practice. WLB initiatives are defined as institutionalised structural and procedural arrangements, both formal and informal, that enable individuals to manage the conflicting work and personal worlds more easily [1]. There are some WLB interventions. As stated by Frone [67], they vary depending on the type of organisation, its size, its subject of activity, and the demographic structure of its employees. Before implementing WLB measures, it may be recommended to incorporate a gender audit to help assess the work environment, organisation culture and employee needs and place them in the context and setting of the organisation, including finding the optimal solution that suits all parties. WLB initiatives include flexible working arrangements (e.g., telecommuting, compressed workweek and flexible working hours), leave arrangements (e.g., maternity leave, paternity leave, leave to care for a sick dependent), dependent care (e.g. daycare, subsidised daycare, elder care, and child care referrals), and general services (e.g., employee assistance programs, seminars, and family needs programs). According to Dhas [55], some WLB programs help employees manage stress, while other programs help reduce absolute stress levels by restoring WLB.

The empirical evidence indicates that reducing the working time of full-time employees, combined with minimum working time guarantees for part-time employees, can lead to many positive impacts for both employees and organisations, including society as a whole: fewer occupational health problems, reduced healthcare costs, more and better jobs, better WLB, more satisfied, motivated and productive employees, leading to more sustainable organisations. In addition, reducing working hours can significantly contribute to the greening of economies, as the more we work, the bigger our carbon footprint is. Thus, reducing the number of working days and, therefore, the time spent commuting to work can reduce the carbon footprint and positively impact the environment. A return to the historic path of shorter working hours, combined with a balanced working time distribution, can also be another step on the long journey towards a healthier life and a more sustainable society [2]. According to the ILO [41], in 2021, the COVID-19 weakened the economic, financial and economic spheres, affecting the labour market and social structure in almost all countries, regardless of the region's level of development. The ILO [41] comprehensively assesses how the global labour market recovery reflects different countries' approaches to addressing the COVID-19 crisis. It analyses global patterns, regional differences and outcomes across economic sectors and groups of employees [41]. Further, the report also offers labour market projections for 2022 and 2023 [41]. In June 2021, 187 ILO Member States adopted a global call to action for a people-centred recovery from the COVID-19 crisis [68]. However, it does not directly address the WLB concept. At the European level, on June 13, 2019, the Council of the European Union adopted the Directive on WLB for parents and carers and repealing [69], which aims to increase women's participation in the labour market and the use of family leave and flexible working arrangements. The new directive [69] also allows employees to take time off work to care for family members who need help. This legislation will enable parents and carers to balance their work and private lives better. Today's accelerated times, in which we are frequently faced with the phenomenon of burnout syndrome, the ever-increasing number of people suffering from depression or other types of mental illness, but also with the breakdown of the traditional family or the lack of social services, require new solutions, both in the form of a legal and a society-wide response to the problem, as the consequences of overlooking and neglecting this issue can be fatal [70]. The latter two phenomena are also the reasons for the long-term exclusion of carers (especially women) from the labour market. The European Union has acted against such adverse effects through the directive mentioned above, and the Member States are obliged to take appropriate measures to fulfil its purpose.

WLB generally enhances job satisfaction, quality of life, organisational commitment, and work consciousness while lowering turnover intentions [33]. Smith and Gardner [9] cite numerous authors who concur that WLB initiatives are vital for organisations' recruitment and retention strategies. According to Chimote and Srivastava [71], from the organisation's perspective, the benefits of WLB are to reduce absenteeism and turnover, improve productivity and image of the organisation, and ensure employee loyalty and retention. In contrast the employee perspective emphasised that the benefits of WLB are job satisfaction, job security, job autonomy, stress reduction, and improved health.

### 2.4 Post-COVID-19 WLB

According to research by Voorspoels et al. [72] and Vyas [6], the COVID-19 significantly impacts the prioritisation of individual areas affecting WLB and enhancing anxiety among the employees of the organisations. In addition, it has increased employees' desire to efficiently manage their families throughout the COVID-19 while continuing to work [73]. Home office, seen as a benefit that helps balance work and personal life, is less attractive after the COVID-19. For example, 56 % of respondents in the research stated that it is hard to switch off after work due to the long-lasting ‘forced’ home office, and 67 % of employees felt less connected to their colleagues [74]. Further, flexible working hours interfere negatively with the life area. The biggest challenge will, therefore, be to evaluate existing research and the results of which can be fundamentally affected by the pandemic two years from now [[75], [76], [77], [78], [79], [80]]. The first results show that for employees, where employers are trying to save money by allocating people to the home office because, during the COVID-19, these conditions were forcibly created, problems are starting to arise in their personal lives, where stressful situations occur as part of the intermingling of work and personal life in one place [80], or health problems occur because people often work from the couch or bed at home [74], thereby disrupting the health in the life area. Many researchers focus on the implications, challenges, and opportunities for HRM brought about by the COVID-19 [[81], [82], [83], [84], [85], [86], [87]].

### 2.5 Social media, social media analysis and Twitter as a research platform

Social media can be characterised as a subset of media that uses internet connections and can also be referred to as ‘new media’ or ‘media 2.0’ [88]. According to Myslivcová et al. [89], the definition of social media is not uniform, it has evolved, and different authors have different perspectives.

Social media platforms like social networking websites are notable for sharing media, updating profiles, facilitating individual connections, and enabling participation in various online communities [90]. Boyd and Ellison [91] defined social network sites as *‘web-based services that allow individuals to (1) construct a public or semi-public profile within a bounded system, (2) articulate a list of other users with whom they share a connection, and (3) view and traverse their list of connections and those made by others within the system’*.

Social networks are a specific social medium that allows everything to be shared. , users share their photos, links, videos, personal information, moods, and current feelings [92]. Generally, they can be defined as a group of individuals who know each other and communicate individually or in groups with a clear goal [88].

A blog is one of the essential elements of social media. It is a website where one regularly posts, displayed chronologically from newest to oldest. Setting up, maintaining, and contributing to a blog is called blogging. The posts are called articles posted by the blogger [93]. Usually, a blog is focused on a particular topic. A special kind is a microblog (e.g. Twitter) where the number of characters is limited. Twitter is an online communication platform, which is technically called ‘microblogging’. The author writes their message (called a tweet) in the appropriate window and sends it. Those who follow them (called followers) can immediately read and respond to their opinions. What is exciting and valuable is the speed at which new information spreads [94]. Since its inception in 2006, Twitter has gained popularity and become very important in communication, often called the ‘SMS of the Internet’. Kvasničková Stanislavská et al. [95] state that Twitter is one of the most popular social networking platforms, used by 329 million monthly active users worldwide [96].

The rise in Twitter's popularity and people's acceptance of it has created an opportunity for more research on this social media [97]. According to Sarlan et al. [98], social media is getting more attention nowadays. Its role as a contemporary communication tool, extends its utility as a valuable instrument for comprehending and expanding the theoretical underpinnings of diverse domains. Public and private opinions on various topics are constantly expressed and disseminated through social media. Twitter provides a quick and efficient way to analyse the opinions of its users. Yue et al. [99] discuss the aspects of sentiment analysis and state that the feelings or views of social media users provide the most up-to-date and broadest information because of the prevalence of social media and the low barrier to message publication. For instance, from an HRM viewpoint, social media analysis might present a fresh and valuable approach through which unprecedented insights can be uncovered, allowing us to uncover contemporary contemporary dynamics and enrich theoretical foundations.

For many employees, social media can be a powerful communication tool that helps them collaborate, share ideas, and solve problems [15]. As addressed by Sharma and Sudhesh [100], the use of social media has become widespread in recent years, and as more and more people connect through online media, it is also essential to find a balance between virtual and real life. The corporate world requires a balance between work and life priorities. Employees often struggle to manage the various responsibilities in their lives, both inside and outside of work. Technological advancements have changed the nature of work and blurred the lines between work and life commitments [100]. The intersection of work and home has received considerable research attention in recent years, with recent research highlighting how virtual telecommuting, supported by sophisticated ICTs, contributes to a reduction in WLB for many employees [101]. Thus, obtaining information from social media platforms can provide valuable insights for theory and practice to better understand the changing needs of employees and organisations and bring new insights for HRM.

Twitter's escalating prominence and widespread acceptance have paved the way for augmented scholarly inquiry into this social media platform [97]. Over the past decade, Twitter evolved into a pivotal medium for academic research, as evidenced by its utilisation of over ten thousand scholarly articles [102]. This was primarily due to Twitter's capability to furnish voluminous, real-time textual datasets that reflected public opinion on various topics [103]. Additionally, Twitter served as a valuable tool for identifying and monitoring prevailing trends [104], as well as for conducting sentiment analysis [105]. This paradigm shift towards incorporating Twitter in academic research highlights the platform's utility in capturing the nuances of public discourse and sentiment, offering researchers a dynamic and rich data source for diverse analytical pursuits.

Social media analysis has been used in many areas of research interest, such as the COVID-19 [106], food [107], corporate social responsibility [95], greenwashing [108], zero waste [109], nursing education [110], burnout syndrome [111] and others.

## 3 Materials and methods

**Fig. 1:**
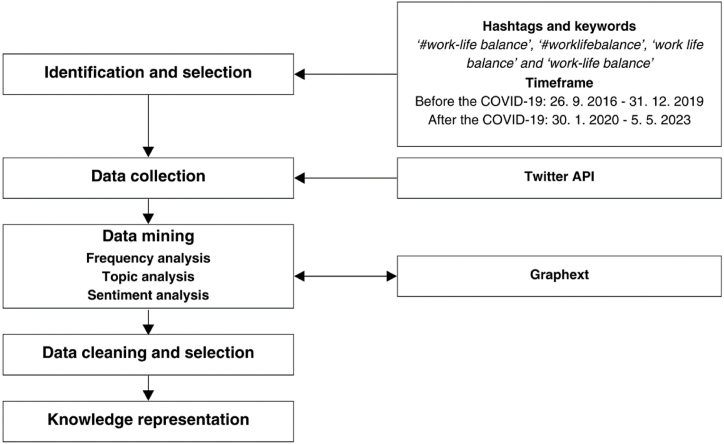
Five steps of the SMAHR framework.

Source: Own elaboration using draw.io software (version 24.2.2)

The methodology used in this research was based on the Social Media Analysis based on the Hashtag Research (SMAHR) framework, which was systematically outlined by Pilař et al. [112]. Our research proposes an innovative approach to HRM research through social media analysis. It allows the capture of unfiltered insights from Twitter discussions, promoting the HRM research landscape by leveraging big data to promote HRM's future practice. The comprehensive SMAHR framework guides the analytical process of this research, consisting of five interconnected stages, as illustrated in Fig. 1.

### 3.1 Identification and selection

The initial stage involves the meticulous identification and selection of relevant hashtags and keywords, which will identify the tweets that will be downloaded from the Twitter social network. Part of this stage is defining the timeframe for which the analysis will be performed. This is crucial for ensuring that the subsequent analysis is grounded in pertinent and meaningful data, thereby contributing to the overall robustness and validity of the research's findings.

#### 3.1.1 Identification and selection of relevant hashtags and keywords

Based on theoretical background, the following hashtags and keywords were selected: ‘#work-life balance’, ‘#worklifebalance’, ‘work life balance’ and ‘work-life balance’.

#### 3.1.2 Timeframe

When conducting research, choosing an appropriate date range is crucial to ensure accurate and relevant findings. In researching WLB communication on Twitter before and after the COVID-19, it is important to consider key events and declarations made by authoritative bodies such as the World Health Organization (WHO). Based on the information provided, the after-COVID-19 timeframe is defined as January 30, 2020, to May 5, 2023 (three years, three months, five days excluding the end date).

On January 30, 2020, the WHO [113] declared a Public Health Emergency of International Concern (PHEIC) in response to the escalating the COVID-19 situation. This declaration signalled the severity of the situation and the need for immediate global attention and coordinated efforts to control the spread of the virus. Therefore, including this date as the starting point of the after-COVID-19 timeframe is appropriate. On May 5, 2023, the WHO Emergency Committee on the COVID-19 [113] recommended to the Director-General that the pandemic, which had been ongoing for more than three years, no longer fit the definition of a PHEIC. This indicates that the situation had become well-established by this date, and efforts had been made to manage its impact. Therefore, including this date as the endpoint of the after-COVID-19 timeframe captures the pandemic's current status as the WHO recommended.

To maintain a consistent timeframe for both the before-COVID-19 and the after-COVID-19 timeframes, we chose a timeframe that matches the three-year, three-month and five days duration of the after-COVID-19 timeframe while also considering the relevant information from the WHO. Based on the data, a significant event occurred on December 31, 2019, when the Wuhan Municipal Health Commission in China reported a cluster of pneumonia cases in Wuhan, Hubei Province, eventually identified as a novel coronavirus [114]. Therefore, this date is a crucial endpoint for the research before the COVID-19. Considering this, the chosen date range for the before-COVID-19 timeframe is from September 26, 2016, to December 31, 2019, excluding the end date. By selecting September 26, 2016, as the starting point, the research encapsulates the years leading up to the emergence of the novel coronavirus outbreak in Wuhan. This allows a comprehensive analysis of the communication of the WLB topic on Twitter during the period before and after the COVID-19.

#### 3.1.3 Selection criteria for user profiles

In our Twitter analysis focused on the topic of WLB, we deliberately chose not to limit our examination solely to verified profiles on Twitter, of which only 14,133 were identified among a total of 499,574 users. The primary motivation behind this decision was to ensure data representativeness, as verified profiles typically represent a specific user group from public life, media, and politics and may not fully reflect the broader spectrum of opinions and experiences associated with WLB. We aimed to include a wide socio-economic and geographical diversity, which verified accounts might restrict. This diversity is crucial for understanding the varied perspectives and experiences within the WLB theme. By incorporating a broad range of users, we also sought to capture the different types of discussions and the dynamics of social media, which are important for a comprehensive understanding of how the topic of WLB manifests in the wider public discourse. This approach ensured that our analysis provided a more comprehensive and layered view of WLB in the public discourse. The last reason is that Twitter stopped user verification from November 2017 until May 2021.

### 3.2 Data collection

Once the relevant hashtags and keywords have been identified and selected, the second stage encompasses systematic data collection. This involves extracting comprehensive datasets from various social media platforms, ensuring a diverse and representative sample that reflects online interactions' complexities and multifaceted nature. In this case, the social network Twitter was selected, which at the time of data collection (May 21, 2023), allowed all data to be obtained via the Twitter Application Programming Interface (API) [115] based on a search command. The Tractor software [116] was used to access the API with the following query: *‘#work-life balance’ OR ‘#worklifebalance’ OR ‘work life balance’ OR ‘work-life balance’*. Based on this, 1,768,628 tweets were downloaded (specifically 915,402 before the COVID-19 and 853,226 after the COVID-19).

### 3.3 Data mining

Following the data collection, the third stage delves into an in-depth analysis of the gathered data Utilising a range of analytical tools and techniques, this stage aims to uncover underlying patterns and themes, providing valuable insights into the dynamics of social media over time and their implications.

The following methods were used: frequency analysis, topic analysis and sentiment analysis.

#### 3.3.1 Frequency analysis

This analysis constitutes a fundamental method utilised to explore the recurrence of specific elements within a given dataset, herein focused on the incidence of hashtags and keywords within a collection of tweets. The primary objective of performing a frequency analysis in the context of social media content, such as Twitter, is to ascertain and quantify the prevalence and popularity of particular hashtags and keywords, thereby facilitating a discernible understanding of prevailing themes, topics, or trends circulating within the platform. This analysis was performed in the Graphext software [117] in the ‘extract hashtags’ and ‘extract keywords’ modules.

#### 3.3.2 Topic analysis

This method is utilised to discern key subjects or themes conveyed within a large dataset, such as posts on social media platforms. Within the multifaceted networks that characterise social media environments, specific nodes (for instance, words or hashtags) demonstrate a higher degree of mutual interconnectedness than other elements within the network. Thus, identifying topics by examining the clusters formed by specific hashtags becomes viable. This stage intended to elucidate the thematic construct of discussions, with a particular focus on WLB, occurring on Twitter. Unlike the frequency analysis approach, which focuses primarily on hashtags, topic analysis in this research was conducted by considering full tweets. Graphext software was engaged for this analysis. To examine the community structure inherent in our network, Graphext employed an adapted version of the Louvain algorithm [118]. The network was shaped based on the interlinkages amongst individual words contained within the tweets. The Louvain algorithm invokes an iterative process that assigns nodes to clusters to optimise a performance metric termed modularity. This metric assesses the relative density of edges within clusters versus those that connect different clusters. Determining distinct communities within the dataset proceeded as specified in Eq. (1).

(1) $$\Delta Q=\left[\frac{\sum_{in}{+2k}_{i,in}}{2m}-{\left(\frac{\sum_{tot}{+k}_{i}}{2m}\right)}^{2}\right]-\left[\frac{\;\sum_{in}}{2m}-{\left(\frac{\sum_{tot}}{2m}\right)}^{2}\right]$$

where $\sum_{in}$ is the sum of weighted links inside the community, $\sum_{tot}$ is the total number of weighted connections inside the community, $k_{i}$ is the total number of weighted links related to the community hashtags, and i, $k_{i,in}$ is the total weighted linkages from an individual to the community hashtags, and m is the normalisation factor, calculated as the total weighted links over the entire graph [118].

#### 3.3.3 Sentiment analysis

The objective underlying the employment of sentiment analysis, particularly within the confines of the Twitter social media platform, is to ascertain the prevailing mood or opinion encapsulated within tweets about a specified topic. Sentiment analysis encompasses a systematic process through which textual content is categorised into respective sentiments (positive, negative, or neutral) based on the context and tonality of the language employed. This procedural step aimed to identify and subsequently analyse sentiments being conveyed in tweets regarding topics related to WLB on Twitter, facilitating a deeper understanding of public perception and discourse about this area of initiatives and discussions. The ‘extract sentiment’ module in the Graphext software was used to identify the sentiment of individual messages.

### 3.4 Data cleaning and selection

After data collection, the data was cleaned. Out of the 162,832 unique before and after the COVID-19 WLB hashtags, the 25 most frequently used ones were chosen for further analysis (see Table 2). However, the most frequent hashtag, ‘#worklifebalance’ (specifically with a total count of 237,900 before the COVID-19 and 159,700 after the COVID-19), ranked first position, was excluded. This is primarily because the hashtag has been the subject of a search and may not provide relevant data for analysis. Out of the 1,365,823 unique before and after the COVID-19 WLB common words, the 25 most frequently used ones were chosen for further analysis (see Table 3). However, some of these common words were excluded mainly because they were related to the search's subject, and some were meaningless (see Table 1). Excluding the hashtag and some common words from the analysis ensures obtaining more relevant and valuable data and avoids biased results or unnecessary noise.

**Table 1:** Excluded common words.

Before the COVID-19			After the COVID-19		
Position	Word	Total count	Position	Word	Total count
1	life balance	444300	1	worklifebalance	157400
2	worklifebalance	235700	2	work life	98830
3	work life	86910	3	work life balance	87630
4	work life balance	76750	16	pisce	38310
8	amp	50230

**Table 2:** The 25 most frequently used hashtags related to WLB on Twitter before and after the COVID-19 between 2016 and 2023.

Before the COVID-19			After the COVID-19		
Position and hashtag		Total count	Position and hashtag		Total count
1	#work	23,670	1	#workfromhome	13,340
2	#balance	19950	2	#work	12,830
3	#worklife	14,140	3	#remotework^a^	11,390
4	#business	12,170	4	#productivity	11,390
5	#productivity	12,010	5	#worklife	9924
6	#life	11,940	6	#leadership	9734
7	#entrepreneur	11,560	7	#mentalhealth	9301
8	#leadership	10,390	8	#wfh^a^	8702
9	#career	9399	9	#balance	8444
10	#hr	8341	10	#jobs	7289
11	#wellbeing	6839	11	#futureofwork^a^	7268
12	#flexibleworking^a^	6633	12	#motivation	6820
13	#workfromhome	6547	13	#wellbeing	6583
14	#success	6514	14	#entrepreneur	6304
15	#startup^a^	6262	15	#business	6103
16	#jobs	5583	16	#life	5737
17	#stress^a^	5390	17	#timemanagement	5634
18	#mentalhealth	5330	18	#selfcare^a^	5095
19	#motivation	5207	19	#success	4843
20	#family^a^	4954	20	#career	4654
21	#health^a^	4951	21	#hr	4632
22	#job	4950	22	#workingfromhome^a^	4619
23	#entrepreneurship^a^	4765	23	#hiring^a^	4503
24	#timemanagement	4570	24	#job	4293
25	#wellness^a^	4550	25	#covid19^a^	4261

a unique values.

**Table 3:** The 25 most common words that appear in the tweets related to WLB on Twitter before and after the COVID-19 between 2016 and 2023.

Before the COVID-19			After the COVID-19		
Position and word		Total count	Position and word		Total count
1	time	70,390	1	time	76,490
2	find	61,170	2	healthy	57,250
3	good	50,910	3	day	50,540
4	day	42,540	4	find	48,340
5	tip^a^	40,130	5	good	46,030
6	help	39,990	6	home	44,420
7	job	39,000	7	job	43,720
8	achieve	39,000	8	healthy work^a^	43,510
9	business^a^	37,170	9	help	40,390
10	family	36,830	10	achieve	40,160
11	great	35,770	11	well	39,420
12	need	35,060	12	need	38,790
13	well	34,830	13	employee	37,480
14	new	31,300	14	new	37,440
15	career	30,470	15	come^a^	35,820
16	employee	28,160	16	great	33,340
17	want	27,100	17	like	32,420
18	thing^a^	26,990	18	people^a^	32,330
19	look	26,600	19	focus^a^	32,020
20	home	26,440	20	look	30,550
21	way^a^	26,270	21	want	29,670
22	like	26,220	22	family	29,540
23	healthy	25,570	23	career	29,140
24	entrepreneur^a^	25,270	24	play^a^	29,130
25	today^a^	24,530	25	week^a^	28,570

a unique values.

### 3.5 Knowledge representation

The procedure of knowledge representation, intrinsically intertwined with the realm of data analytics, capitalises on visualisation tools to illuminate the findings unveiled through data mining endeavours lucidly. This process emphasises the amalgamation of discrete values and consequential outcomes derived during the data appraisal phase, converting data into understandable, actionable insights. The main objective of this stage is to meticulously extract, highlight, and subsequently communicate pivotal findings originating from preceding analyses, ensuring that data-driven discoveries are rendered accessible and intelligible to diverse stakeholders. This encompasses data representation in a visually digestible format and the discernment and communication of underlying patterns, trends, and anomalies identified through previous investigative stages. Knowledge representation serves not merely as a conduit for data presentation but also as a mechanism for facilitating an enhanced understanding of the data and ensuring that the insights garnered therein can be proficiently utilised to inform decision-making processes and strategic directions.

## 4 Results and discussion

### 4.1 Hashtags and common words

Table 2 shows the most frequently used hashtags related to WLB on Twitter before and after the COVID-19 between 2016 and 2023. Analysis of the top 25 hashtags revealed some changes in the communication of the used hashtags on Twitter.

The hashtags #work (1), #balance (2) and #worklife (3) are the most frequently occurring before the COVID-19. These hashtags are closely related to the terms directly searched. The top ten occurrences include hashtags primarily related to entrepreneurship, productivity and areas falling under the domain of HRM, #business (4), #productivity (5), #entrepreneur (7), #leadership (8), #career (9), #hr (10), with #life in 6th place. No unique values appear in the top ten hashtag occurrences compared to those communicated after the COVID-19. After the COVID-19, the most frequently occurring hashtag is #workfromhome (1), while before the COVID-19, it held the 13th position. This is followed by #work (2) and a completely new hashtag that only appeared after the COVID-19, #remotework (3). This is in line with the changes that took place in response to the restrictions related to the COVID-19. Furthermore, hashtags that also appeared before the COVID-19, such as #productivity (4), #worklife (5) and #leadership (6), occur most frequently in the top ten after the COVID-19 ones. The #mentalhealth hashtag appears in 7th place (18th before the COVID-19), so there is a noticeable shift in the communication and importance of this topic. Rounding out after the COVID-19 top ten hashtags are #wfh (8), #balance (9) and #jobs (10). The second top ten hashtags before the COVID-19, ranging from 4550 to 6839 occurrences, contain hashtags about mental well-being, health, stress, flexible working hours, motivation, work, etc. After the COVID-19, hashtags ranging from 4261 to 7268 occurrences that were in the top ten before the COVID-19 appear in the second ten occurrences, e.g. #entrepreneur (14), #business (15), #life (16), #career (20), #hr (21).

We can identify unique hashtags before the COVID-19 that do not occur after the COVID-19. These are the hashtags #flexibleworking (12), #startup (15), #stress (17), #family (20), #health (21), #entrepreneurship (23) and #wellness (25). Conversely, unique hashtags such as #remotework (3), #wfh (8), #futureofwork (11), #selfcare (18), #workingfromhome (22), #hiring (23) and #covid19 (25) appear after the COVID-19. It is possible to observe the significant influence of the COVID-19 on WLB's communication on Twitter in the context of working from home (WFH), new forms of flexible working and the impact on recruitment and HRM practices.

As part of the WLB's Twitter communication analysis, we also looked at the most frequently occurring words in the individual tweets. Table 3 shows the top 25 most common words appearing in tweets about WLB communication.

In the period before and after the COVID-19, the word time (1) is the most frequent in tweets. Before the COVID-19, words such as find (2), good (3), day (4) and tip (5) were after the COVID-19 are replaced by healthy (2), day (3), find (4) and good (5). Unique words before the COVID-19 are tip (9), business (9), thing (18), way (21), entrepreneur (24), and today (25), and after the COVID-19 are healthy work (8), come (15), people (18), focus (19), play (24), and week (25). It is noticeable that communication before the COVID-19 concerning WLB was more focused on the area of work and presence (words such as today, job, business, career, employee, entrepreneur), although after the COVID-19, some of these words also occur (job, employee, career), there is a higher incidence of words related to the topic of health (healthy, healthy work). Thus, after the COVID-19, the communication of WLB in the organisational setting remains, but there is more communication of the focus on employees in the context of a healthy workplace, which are undoubtedly areas significantly affected by the COVID-19.

### 4.2 Topics and sentiment

Topic analysis provides a different method for analysing communication on social networks. The topic analysis allows a better understanding of the overall dynamics of communication by identifying connections between hashtags. Table 4 and Table 5 show the results of the topic analysis, which identifies topics within Twitter communication related to WLB search terms before and after the COVID-19.

**Table 4:** The topics detected in connection with the WLB hashtag on Twitter before the COVID-19 between 2016 and 2019.

Before the COVID-19		
Topics and key terms	Selection count	Percentage
*Performance (1)* leadership, success, team, office, business, company, productivity, achieve, career, employee	193,300	21.11 %
*Time management (2)* time, hour, day, productivity, schedule, email, think, trying to balance, spend, better work	132,600	14.49 %
*New knowledge (3)* read, article, think, find, blog, know, new, quote, myth, people	37,760	4.12 %
*Parenthood (4)* parent, mom, family, mother, parenting, dad, working parents, motherhood, family life, parental	37,490	4.10 %
*Gender equality (5)* woman, women, female, gender, talk, career, family, man, business, achieve	36,640	4.00 %
*Beginnings of business (6)* entrepreneur, business, owner, startup, smallbusiness, business owners, smallbiz, success, founder, manage	27,190	2.97 %
*Workplace flexibility (7)* employee, flexible, employer, company, flexible working, flexibility, workplace, job, worker, satisfaction	26,830	2.93 %
*Mental health prevention (8)* stress, burnout, avoid, burn, avoid burnout, anxiety, prevent, mentalhealth, stressfull, stressmanagement	16,690	1.82 %
*Healthcare (9)* nurse, physician, care, doctor, medical, patient, medicine, healthcare, hospital, practitioner	15,010	1.64 %

**Table 5:** The topics detected in connection with the WLB hashtag on Twitter after the COVID-19 between 2020 and 2023.

After the COVID-19		
Topics and key terms	Selection count	Percentage
*Recruitment (1)* look, join, new, hire, benefit, remote, offer, career, job, find	132,000	15.47 %
*Employee development (2)* people, important, job, career, company, employees, grow, focus, help, business	124,100	14.54 %
*Time (3)* day, hour, week, time, sleep, weekend, schedule, break, start, spend	98,880	11.59 %
*Leisure (4)* holiday, vacation, break, summer, enjoy, rest, fun, wellness, friend, family	54,240	6.36 %
*Women talks (5)* woman, podcast, episode, gender, female, discuss, interview, series, talk, listen	40,650	4.76 %
*Remote work (6)* home, remote, remote work, work from home, hybrid, remote workers, space, remotely, office, commute	32,640	3.83 %
*Mental health prevention (7)* burnout, stress, mental, anxiety, avoid, mindfulness, stressmanagement, care, meditation, mental health	25,670	3.01 %
*Parenting and relationships (8)* teacher, parent, family, mom, school, kid, dad, parenting, caregiver, working parents	25,540	2.99 %

**Fig. 2:**
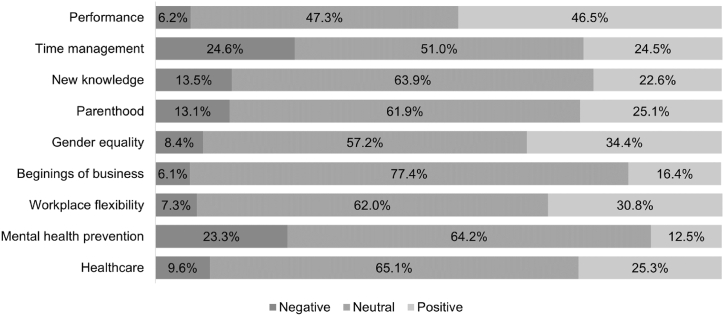
Results of the sentiment analysis on Twitter before the COVID-19 between 2016 and 2019.

Source: Own elaboration according to analysis performed in the Graphext software using Microsoft 365 Excel (version 2402)

**Fig. 3:**
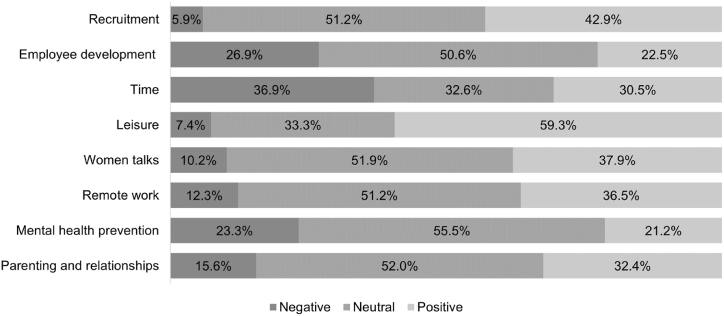
Results of the sentiment analysis on Twitter after the COVID-19 between 2020 and 2023.

Source: Own elaboration according to analysis performed in the Graphext software using Microsoft 365 Excel (version 2402)

Sentiment analysis is a valuable tool for processing social media data and allows the detection of positive, negative or neutral sentiment hidden in users’ posts. Fig. 2 shows results of the sentiment analysis on Twitter before the COVID-19 between 2016 and 2019 and Fig. 3 shows the results of the sentiment analysis on Twitter after the COVID-19 between 2020 and 2023.

#### 4.2.1 Before the COVID-19

A total of 9 significant topics are identified in the period before the COVID-19: *Performance* (1), *Time management* (2), *New knowledge* (3), *Parenthood* (4), *Gender equality* (5), *Beginnings of business* (6), *Workplace flexibility* (7), *Mental health prevention* (8) and *Healthcare* (9). The most significant themes are the first two identified: *Performance* (21.11 %) and *Time management* (14.49 %). The remaining seven identified topics do not exceed the 100,000 selection count threshold.

The largest identified topic is *Performance* (193,300), which includes aspects of business, performance, or efficiency: company, success, productivity, leadership, career, etc. Employee WLB affects performance. When WLB is at a reasonable level, it affects employee satisfaction and performance. A satisfied and motivated employee equals full use of their work potential, productivity growth, and organisation productivity [[119], [120], [121]]. On the other hand, people who communicate about WLB concerning organisation and performance may express that performance requirements may negatively affect their WLB [[122], [123], [124]].

The second largest communicated topic is *Time management* (132,600). The topic comprises concepts that relate to aspects of time such as time, hour, day, and concepts such as schedule, productivity, email, think, trying to balance, spend, or better work. WLB refers to the time people spend at work or at home and when they spend it that way. Whether it is at the weekend, or how many hours are worked during the working week and how efficiently that time is used, has an impact. The proper management of time, planning activities, appropriate scheduling of working hours, etc., influences WLB. Time management moderates the relationship between work overload and work performance, making the relationship between these two factors less negative [125]. Time management has a significant positive effect simultaneously and partially on employees’ WLB [126].

As mentioned, the remaining seven identified topics do not exceed the 100,000 selection count threshold. The *New knowledge* topic (37,760) is characterised by terms like read, article, think, find, blog, know, new, quote, myth, and people. Various means and media can be used to communicate and disseminate awareness and knowledge about WLB through which information and knowledge are shared, information is sought, and myths are debunked. Topic *Parenthood* (37,490) includes terms like parent, mom, family, mother, parenting, dad, working parents, motherhood, family life, and parental. The topic of WLB is likely addressed by people at the stage of parenthood when the balance between work and family is significantly disrupted, and the parent is in a situation where they are looking for the right balance of time devoted to work and family to keep them and the immediate family happy. The *Gender equality* topic (36,640) is defined by terms like woman, women, female, gender, talk, career, family, man, business, and achieve. The issue of gender equality is related to WLB. According to this generated topic, it is evident that Twitter users often communicate the topic of WLB regarding gender issues, women's, and men's rights, etc. The *Beginnings of business* topic (27,190) consist of terms like entrepreneur, business, owner, startup, smallbusiness, business owners, smallbiz, success, founder, and manage. The start-up phase of a business is undoubtedly challenging for both the founder and the employees and impacts WLB. Tahir [127] states that little is known about entrepreneurs' WLB challenges, especially regarding whether entrepreneurship improves individuals' WLB and that some studies have found that the desire for WLB motivates entrepreneurial careers [128] and having one's own business can provide WLB, especially in terms of spending adequate time with family [129]. The *Workplace flexibility* topic (26,830) comprises terms such as flexible working, flexible, workplace, job, worker, and satisfaction. Flexibility in the work environment is one of the factors influencing WLB [130,131]. Topic *Mental health prevention* (16,690) is defined by terms like stress, burnout, avoid, burn, avoid burnout, anxiety, prevent, mentalhealth, stressfull, stressmanagement. In connection with WLB communication, people before the COVID-19 addressed mental health and well-being, stress and its consequences, and prevention, avoiding the adverse effects of stress. The last identified topic is *Healthcare* (15,000) with terms such as nurse, physician, care, doctor, medical, patient, medicine, healthcare, hospital, and practitioner. Due to the demanding nature of the healthcare profession, the topic of WLB is very timely and addressed [[132], [133], [134], [135]].

The analysis of sentiment before the COVID-19 (see Fig. 2) shows that the highest proportion of negative sentiment is caused by the communication of the topics of *Time management* (24.6 %) and *Mental health prevention* (23.3 %) followed by *New knowledge* (13.5 %) and *Parenthood* (13.1 %). The negative sentiment about *Mental health prevention* may not be due to the public's disapproval of the communicated topic but to the generally negative emotions such as sadness and fear that mental health problems evoke. Conversely, the highest levels of positive sentiment can be identified for the topics of *Performance* (46.5 %), *Gender equality* (34.4 %) and *Workplace flexibility* (30.8 %). Overwhelmingly, all topics evoked neutral sentiment, with the highest levels of neutral sentiment for the topic *Beginnings of business* (77.4 %) communicated regarding WLB.

#### 4.2.2 After the COVID-19

A total of 8 significant topics are identified in the period after the COVID-19: *Recruitment* (1), *Employee development* (2), *Time* (3), *Leisure* (4), *Women talks* (5), *Remote work* (6), *Mental health prevention* (7), *Parenting and relationships* (8). The most significant themes are the first two identified: *Recruitment* (15.47 %) and *Employee development* (14.54 %). The remaining six identified topics do not exceed the 100,000 selection count threshold.

The largest identified topic is *Recruitment* (132,000), which includes terms like look, join, new, hire, benefit, remote, offer, career, job, and find. The second largest communicated topic is *Employee development* (124,100), which consists of terms like people, important, job, career, company, employees, grow, focus, and help. Przytuła et al. [136] consider the biggest challenges for HRM in the post-pandemic period to be workplace restructuring, including its content and the use of more advanced technologies in recruitment, selection and performance. They emphasise the importance of managers' appreciation and motivation of employees, building trust and belonging in the team. Benefits should be more focused on promoting mental health and well-being. New competencies are expected for HRM and managers after the COVID-19, requiring retraining and training [136]. Tamunomiebi and Oyibo [137] reviewed the literature on WLB and employee performance and proposed appropriate solutions to overcome the problem of work-life imbalance and its associated negative impacts to enhance employee performance and achieve optimal organisational outcomes. The association between WLB and employee development has also been explored by Deery and Jago [138], Wolor et al. [34] and Aruldoss et al. [139].

As mentioned, the remaining six identified topics do not exceed the 100,000 selection count threshold. Topic *Time* (98,880) is characterised by terms such as day, hour, week, time, sleep, weekend, schedule, break, start and spend. Individual time periods and parts of the day impact the WLB. Topic *Leisure* (54,240) includes terms such as holiday, vacation, break, summer, enjoy, rest, fun, wellness, friend, and family. Well-being, relaxation, vacation, and fun are potential factors influencing WLB. The amount of time people spend at work is an important aspect of WLB. The more people work, the less time they have for personal care or leisure, yet the quantity and quality of leisure time is important for people's overall well-being and has a positive effect on physical and mental health [140]. *Women talks* topic (40,650) is defined by concepts such as woman, podcast, episode, gender, female, discuss, interview, series, talk, and listen. After the COVID-19, as before the COVID-19, the communicated topic is the area of knowledge, media and information dissemination, but according to the characteristic words defining the topic, it is more related to women. The *Remote work* topic (32,640) consists of terms such as home, hybrid, remote workers, space, home, office, and commute. The COVID-19 has significantly impacted how people work, and there has been a marked expansion of so-called flexible work arrangements such as WFH. During WFH psychological detachment, sleep, stress, social support, WLB and productivity are reduced. Psychological detachment significantly affects stress and sleep, which in turn affects productivity. Social support significantly helps to maintain WLB. The key to increasing productivity and WLB during WFH is to promote psychological detachment and social support in employees [141]. The *Mental health prevention* topic (25,670) comprises terms such as burnout, stress, mental, anxiety, avoid, mindfulness, stressmanagement, care, meditation, and mental health. Similarly, as before the COVID-19, in connection with WLB communication, people addressed mental health and well-being, stress and its consequences, and prevention, avoiding the adverse effects of stress. The last identified topic is *Parenting and relationships* (25,540), defined by terms such as teacher, parent, family, mom, school, kid, dad, parenting, caregiver, and working parents. The COVID-19 significantly influenced this domain, and balancing work and childcare was central for parents [[142], [143], [144]].

The analysis of sentiment after the COVID-19 (see Fig. 3) shows that the highest proportion of negative sentiment is caused by the communication of the topics of *Time* (36.9 %), *Employee development* (26.6 %) and *Mental health prevention* (23.3 %). Conversely, the highest levels of positive sentiment can be identified for the topics of *Leisure* (59.3 %), *Recruitment* (42.9 %), *Women talks* (37.9 %), and *Remote work* (36.5 %). The topic of *Mental health prevention* elicits the highest neutral sentiment (55.5 %). Future research should focus on this to comprehensively understand and address the evolving sentiments in post-pandemic contexts, ultimately informing HRM effective strategies. Further, the identified associations between specific topics and negative or positive sentiment offer promising avenues for further research into how these sentiments reflect evolving workplace dynamics and societal changes in the post-pandemic era. Exploring the underlying factors behind these sentiment patterns can deepen our understanding of the pandemic's impact on emotional responses and individual perspectives.

#### 4.2.3 Comparison and discussion of results before and after the COVID-19

For the after-COVID-19 timeframe, there is a predominant focus on HRM communications, such as recruitment and telework, as well as human capital and employees. The recruitment theme constitutes the newly identified topic in the after-COVID-19 timeframe (*Recruitment*, 15.47 %). With the *Employee development* topic (14.54 %) and the *Remote working* topic (3.83 %), the total communication of HRM theme has gained importance after the COVID-19 (33.84 %) and accounts for a third of the WLB-related communication on Twitter in the after-COVID-19 timeframe. During the COVID-19, many people worldwide were forced to work remotely. Initially anticipated as a positive factor supporting WLB, the ability to WFH as a positive factor manifested negative tendencies over time, which caused more stress, and all groups of employees faced difficulties in reconciling work and private life, especially women and people with young children [145]. Women were slightly more likely to lose their jobs during the pandemic than men, and those who remained employed were likelier to WFH [146]. The expansion of remote work in the context of the COVID-19 led to a new geography of work and new spaces of inequality, manifested in significant differences in work experiences and outcomes, particularly by race, generation, and number of dependents [147]. Ferreira et al. [148] mention that introducing of remote working in HRM is increasingly common, either for economic reasons, competitive advantage or in response to the COVID-19. The advantages are cost reduction and flexibility in supporting WLB. On the other hand, communication, technical and management issues can be considered as concerns. However, recognising more positive relationships than negative ones prevails [148]. The COVID-19 has fundamentally changed the world of work, especially recruitment and hiring. According to the European Labour Authority [149], virtual job interviews have become the new standard, location is no longer a deciding factor, there has been a demand shift in the job market satisfying the need for job security, and organisations need to emphasise employer brand and self-attractiveness.

*Time* which undoubtedly impacts WLB, has moved from second place (14.49 %) in the before-COVID-19 timeframe to third place (11.59 %) in the after-COVID-19 timeframe. This is a 2.9 % decrease in topic size. However, time is still a hot topic in both timeframes. The topic of WLB and time management was addressed by Chansaengsee [150], who examined the impact of time management on WLB and study. Using a set of time management programmes and behaviour modification techniques can be considered effective in achieving WLB. Brough et al. [151] looked at definitions of WLB, including ones focusing on the equality of time spent in work and non-work areas, satisfaction with performance and time spent in each area and the importance of each role to the individual. The most significant theme of organisations’ productivity, performance or efficiency (21.11 %) relating to WLB identified before the COVID-19 no longer appears as a separately significant topic in the after-COVID-19 timeframe. Still, the link to organisation growth can be observed in the *Employee development* topic identified after the COVID-19. Ismawati et al. [152] found that WFH positively affects individual emotional well-being and balance, increasing happiness, reducing emotional sensitivity, and increasing time efficiency, including productivity. For WFH to be successful, good management and a balanced approach are required, as well as implementing policies that promote WLB for employees. They emphasise the requirement for flexibility in time, location, work, and leave schedule. WLB has a positive impact on job satisfaction and performance. Job satisfaction partly mediates the relationship between WLB and job performance [153]. In the fourth place in the after-COVID-19 timeframe, the *Leisure* topic (6.36 %) is identified, which did not appear at all in the before-COVID-19 timeframe, so this is a unique occurrence of the importance of communication on the topic of vacation, relaxation, rest concerning WLB harmonisation. The terms are related to rest, relaxation and personal life. It refers to time spent away from work responsibilities where people can enjoy holidays, time off, summer and time with friends, having fun while caring for their physical and mental health. These activities are vital to achieving WLB. Bartlett et al. [154] describe ten rules to improve academic WLB. For example, rule six highlights the importance of taking time to care for oneself and relax in life and rule seven to interact with family and friends. Vacations, free time and work flexibility, as factors related to WLB support, are among the top priorities of millennials, according to ManpowerGroup [155].

In fourth place in the before-COVID-19 timeframe, but with a 2.24 % lower percentage, is the identified topic *New knowledge* (4.12 %). The theme of new knowledge creation and dissemination concerning WLB, but with a greater emphasis on women, occurs in a separate topic in the after-COVID-19 timeframe, namely the topic in fifth place called *Women talks* (4.76 %). The topic of means of communication, choice of media, discussions, and overall transfer of information and knowledge is a theme communicated both before and after the COVID-19. The terms that comprise the content of both topics are related to information, ideas, and communication, which can affect how people cope with their work and personal lives. For example, reading articles, and blogs, searching online for information about work-related issues, or sharing opinions, quotes, etc., may create a need to balance these activities with time spent outside work. Norouzi et al. [156] examined the effect of stress management training using podcasts on work-life conflict and concluded that stress management through podcasts reduced work-life conflict.

The topic of parenting and its link to WLB was also present in Twitter communication in both timeframes but in slightly different forms. In the before-COVID-19 timeframe, a topic called *Parenthood* (4.10 %) was identified that included communication of the influence of mother, father or family on WLB, while in the after-COVID-19 timeframe, a smaller topic called *Parenting and relationships* (2.99 %) is identified that includes not only the theme of family, mother, father or parenting but also more recently the relationship between family and school. The negative impact of work on WLB tends to be concentrated in the early stages of parenthood when employees have pre-school-age children in the household [45]. Barriers to family-school collaboration due to the COVID-19 have been the focus of research by Švaříček et al. [157] or Smetáčková and Štech [158], whose questionnaire mapped families' facilities, parents’ time and competences, teaching progress, parental practices in helping with learning and communication with the school. The topic of the distribution of educational responsibility in the two waves of the COVID-19 in the Czech Republic was investigated by Kaščák et al. [159]. Carrión-Martínez et al. [160] presented critical information on the relationship between the family and the school during the period of distance education caused by the COVID-19. They verified that the education scenario during the COVID-19 was one of the most significant challenges in the recent history of education. Allen and Kiburz [161] provide evidence of the importance of sleep quality for working parents and find that sleep quality is also positively related to WLB. Working parents who report higher trait mindfulness report greater work-family balance and thus better self-regulation, which is associated with mindfulness. This may enable individuals to experience satisfaction and effectiveness within each role. The process by which trait mindfulness is related to work-family balance is through increased sleep quality and vitality. Individuals with greater mindfulness tend to experience greater sleep quality and vitality related to greater work-family balance [161].

In the after-COVID-19 timeframe, there is a separately identified topic devoted to *Gender equality* (4 %), in contrast to before the COVID-19 when there was no such topic. The communication theme of women, gender and female includes the identified topic *Women talks* (4.76 %), which additionally consists of the theme of communication media or media. According to Farre et al. [146], the COVID-19 appears to have increased inequalities between men and women in paid and unpaid work in the short term. Son Hing et al. [162] present a comprehensive review of the extent of gender inequalities in organisational domains and practices, which include areas such as performance appraisal, compensation, leadership, work-family conflict, and sexual harassment across the employee life cycle from selection to leave and propose a Model of Cumulative Gender Inequities in the Workplace.

Communication about mental health, stress, prevention, etc., appears in separately identified topics in both timeframes (topics of *Mental health prevention*). Before the COVID-19 (1.82 %) and after the COVID-19 (3.01 %) and thus an increase in communication on this important topic can be observed after the COVID-19. The enforced lockdown during the COVID-19 did not improve WLB for most people, even though they could spend more time with family and commute time was eliminated [163]. There is a positive association of WLB with work-life satisfaction and a negative association with anxiety and depression in all cultures studied, with individualism or collectivism and gender egalitarianism moderating the relationships. WLB benefits employees from different cultures, and culture mediates these relationships [59]. Singh et al. [164] point out how work and personal digital platforms induce technostress during forced telecommuting, increasing psychological distress such as technological exhaustion and reduced subjective well-being. As a mental health prevention intervention, individuals or organisations can consider mindfulness-based interventions to facilitate employee health, vitality, and work-family balance. Dual approaches that include individual-based methods, such as mindfulness training, and situational methods, such as family-supportive supervision, can be optimally effective. Thus, by understanding affect regulation and how mindfulness contributes to it, new insights can be uncovered about the prevalence of work-family balance, work-family conflict, family enrichment, and health-related behaviours [161]. According to Eurofound [44], at the European level, the proportion of employees reporting difficulties reconciling work and family life has remained stable since 2000 (around 20 %). However, there is considerable variation between countries [44].

Communication of the link to business start-ups and WLB was identified in the before-COVID-19 timeframe (*Beginnings of business*, 2.97 %) but was no longer separately identified after the COVID-19. Similarly, workplace flexibility is identified before the COVID-19 (*Workplace flexibility*, 2.93 %) but not separately identified after the COVID-19. Flexible work arrangements allow employees to schedule activities and use their time more efficiently, thanks to flexible working and teleworking [165]. The *Healthcare* topic (1.64 %) and WLB linkages were communicated in the before-COVID-19 timeframe, while it was no longer identified after the COVID-19.

Consistently in both timeframes, the temporal aspect of WLB and the topic of mental health and its prevention are negatively perceived. In the after-COVID-19 timeframe, Twitter communication is newly negatively associated with HRM and WLB. This is presumably because the COVID-19 has dramatically affected and impacted HRM approaches as mentioned and cited above. But recruiting, one of the HRM activities, has seen a positive sentiment after the COVID-19. Thus, it would be interesting to identify what specific activities falling under HRM are negatively communicated and to find out the reasons for the positive communication of the topic of recruitment.

Based on the results, we consider HRM in the context of WLB support as a key process that covers concrete practices that organisations should use to actively create and maintain a balance between employees’ work responsibilities and their personal life needs. HRM should aim to identify, optimise, and implement policies, programmes and processes that enable employees to achieve a WLB. Such practices include recruitment and selection strategies that respect the need for flexible working environments and support teleworking, programmes to improve time management, employee mental health programmes, programmes to support women in the workplace, and parenting and family care.

#### 4.2.4 Towards the proposed post-pandemic definition of WLB in the HRM context

According to the theoretical background, there are several definitions of WLB. Still, over the years, WLB has become a contradictory term with several meanings, and to date, there is no universally accepted definition [[52], [53], [54]]. However, the concept of WLB is widely used [166]. Recognising the research's limitations, such as using Twitter as a single source of data, we propose a new definition and concept for an integrated model in the context of HRM designed to support WLB in a unique post-pandemic environment.

**Fig. 4:**
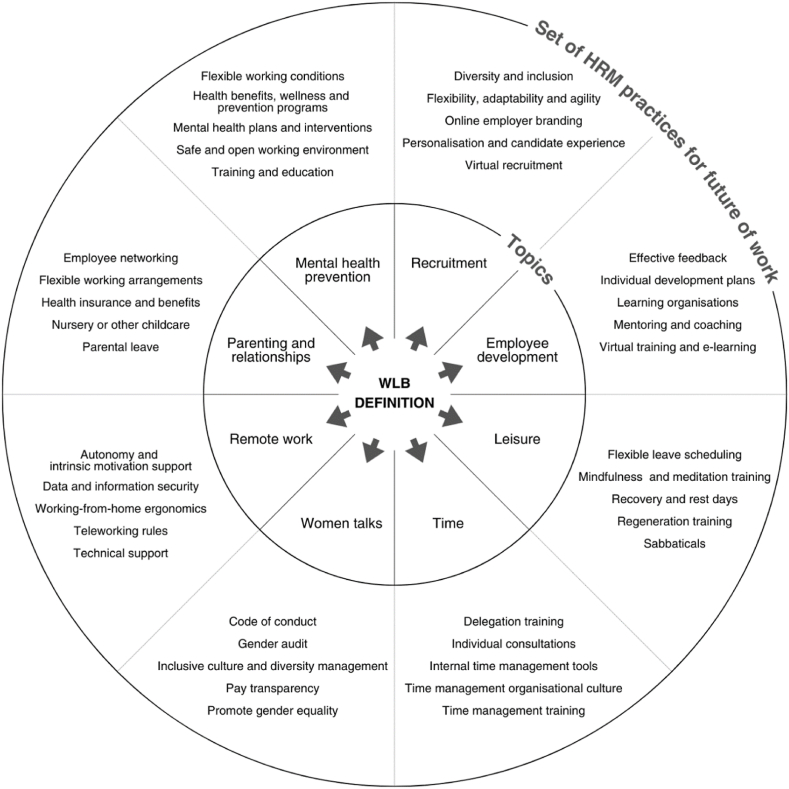
Integrated WLB model within HRM and future of work.

Source: Own elaboration using Canva software (version 1.84.0.0)

Based on the social media analysis, we identified eight significant topics after the COVID-19 (*Recruitment*, *Employee development*, *Time*, *Leisure*, *Women talks*, *Remote work*, Mental health prevention, and *Parenting and relationships*). Most topics communicated concerning WLB relate to HRM practices and the future of work. These topics that people communicate in the WLB context are crucial to them and impact their work-life satisfaction. WLB is a multifaceted concept that has several vital topics emerging from Twitter discussions. In line with that, we propose a new definition**:** ‘*Work-Life Balance in Human Resource Management after the COVID-19 pandemic is a comprehensive concept encompassing the set of practices that organisations implement to help employees manage their work and personal commitments in a way that promotes their overall life satisfaction.*’ This definition reflects current trends and, in the case of a more robust research approach such as future research possibilities, could represent a strategic pillar for effective HRM and the future of work with respect to the challenges and opportunities of the modern workplace, with an emphasis on mental health, sustainability and overall employee well-being. We also suggest an integrated model of key HRM practices promoting WLB (see Fig. 4).

In the middle of Fig. 4 lays the proposed definition of WLB. Around the definition are the identified topics and the HRM practices organisations should implement to promote WLB. These HRM practices play a crucial role in the modern organisation environment, which is increasingly evolving towards flexibility, technological advancement, and an emphasis on employee well-being. They ensure that organisations respond effectively to their employees’ challenges and needs. They aim to enhance the performance and competitiveness of the organisation while at the same time being mindful of employee well-being and ethical principles. The importance of these practices lies primarily in improving productivity and performance, enhancing employee satisfaction, keeping abreast of current trends, and promoting diversity and inclusiveness.

The integrated WLB model brings fresh insights into WLB in the realm of HRM and future of work. It provides a multi-purpose tool for various purposes, such as summarising research findings. Furthermore, it facilitates in-depth discussions on the relationships among different facets of WLB, fostering a deeper understanding of their interconnected dynamics. Beyond research applications, this integrated WLB model offers possible practical utility. It can be employed as an educational resource to enlighten employees about the significance of WLB, thereby promoting a culture of balance within the organisation. Additionally, it serves as a cornerstone for building a positive employer brand, projecting an image of an organisation that prioritises the well-being of its workforce. Importantly, this adaptable integrated WLB model allows customisation to suit the specific needs of different organisations or research contexts. By helping employees achieve WLB, HRM plays a pivotal role in cultivating a more positive, productive, and harmonious work environment. This integrated WLB model underscores the importance of HRM in achieving these organisational goals and enhancing the overall quality of work-life for employees.

Considering the results from different perspectives can deepen the understanding of the main conclusions and can provide useful information for various stakeholders. Our research underscores the urgent need for policymakers to reconsider traditional employment regulations and policies considering the post-pandemic shift towards remote work and flexible work arrangements. Implementing workplace policies that support mental health, encourage work-life integration, and facilitate remote work can enhance employee well-being and productivity. Governments should consider incentives for organisations that adopt innovative WLB practices, ensuring that the workforce is equipped to meet future challenges. The findings from our research highlight the societal shift towards valuing mental health and well-being alongside professional achievement. Individuals increasingly seek careers that offer flexibility, work-life integration, and development opportunities. This shift necessitates a broader societal dialogue on redefining success in the workplace, promoting a culture that values well-being as much as productivity. For organisations, our research provides clear evidence of the evolving expectations of employees regarding WLB and mental health support. Businesses that proactively adapt their HRM practices to address these expectations will attract and retain top talent and build a more resilient and satisfied workforce. Investing in employee development, supporting remote work, and creating a culture prioritising mental health are key strategies for organisations aiming to thrive in the post-pandemic world. An approach from different perspectives can enrich our findings and stimulate further discussion and research in this area.

Our research, conducted on Twitter data, comes with inherent limitations. Firstly, Twitter users are a self-selected group, limiting the representation of individuals who do not engage with the platform. The research focuses on Twitter data, and the findings may not be directly generalisable to offline or non-Twitter contexts. Moreover, our analysis is based on publicly available tweets, introducing potential sampling bias and skewing the representation of specific demographics or opinions. Additionally, our research focuses exclusively on the discourse surrounding WLB before and after the COVID-19, omitting consideration of other influential events or factors. This specificity may limit the broader applicability of our findings. Moreover, though valuable, sentiment analysis may oversimplify the nuanced emotions and perceptions expressed in the textual data, and the generalisability of our results is restricted to the Twitter context, potentially failing to encompass the full spectrum of global perspectives on WLB. Finally, resource constraints have inevitably influenced the depth and breadth of our research.

We want to emphasise that our research offers insights into the dynamics of communicated topics in the context of WLB before and after the COVID-19 but does not have the ambition to predict future developments accurately. The causal link between the results of the topic analysis and the impetus to redefine the concept and proposed integrated model represents a unique approach to using social media analysis results. Other authors must contrast our approach, and its methodological validity should be demonstrated. We know these limitations and believe our results can serve as a valuable basis for future research. Our article encourages further researchers to define the concept of WLB. Based on the more robust research approach and the validation of our proposed model, a new model could serve as a strategic implication for HRM practices in the future of work.

Notwithstanding these limitations, our research brings valuable insights into WLB discourse on Twitter and provides a foundation for further research of the dynamic relationship between WLB and HRM. Future research can build upon our findings to enhance and create a more comprehensive understanding of WLB across diverse contexts.

## 5 Conclusions

This research used a social media data analysis to identify key characteristics of WLB communication on the global Twitter platform before and after the COVID-19. We have examined the evolution of communication regarding WLB. The analysis revealed that after the COVID-19, numerous topics related to HRM and work and personal life management have taken centre stage. Key topics included *Recruitment*, *Employee development*, *Time*, *Leisure*, *Women talks*, *Remote work*, *Mental health prevention* and *Parenting and relationships*. These topics reflect the new modern workplace's challenges and underscore the importance of mental health, sustainability, and overall employee well-being.

Based on the results of the analysis, by proposing a new definition of WLB in the context of the future of work after the COVID-19, we invite academics to redefine the concept. Our proposed definition draws on the themes communicated on Twitter in relation to WLB. It could serve as a basis for effective management considering the opportunities and challenges presented by the contemporary workplace, with a strong focus on employee mental health and well-being. We also proposed the integrated WLB model that outlines key HRM practices to support WLB and the future of work. These practices are essential to address employees' evolving needs and challenges while enhancing organisational performance and competitiveness. They emphasise the importance of employee well-being and ethical principles, which ultimately contribute to a positive workplace culture.

In conclusion, the presented research offers fresh insights into WLB within the future of work and yields several interesting contributions to scientific knowledge. To the best of our knowledge, this research is unique in serving as the first comprehensive examination of the global discourse of WLB on Twitter using big data and, based on the findings, it opens up the possibility for future research approaches by proposing the new definition of the WLB concept, including the integrated model of WLB in the context of future HRM practice and the need for a more robust approach. In examining the characteristics of WLB communication on Twitter, the research uses an automated machine learning approach to automatically analyse the content of tweets instead of using manual coding techniques. It provides a valuable tool for research, facilitates deeper discussions on the dynamics of WLB, and thus presents possible practical applications for organisations seeking to prioritise their employees' well-being. Especially in a rapidly changing work environment, obtaining information from social networks provides valuable insights for theory and practice to enhance HRM practice in the context of future of work. WLB, as a vital element contributing to a more positive, productive, and harmonious work environment, is crucial for HRM's future practice. Understand the changing needs of employees and organisations, the labour market situation and trends faced and confronted by the employment sector in the context of WLB with the HRM's future workplace implications, advance knowledge in the field and provide a basis for further research.

## Ethical approval and informed consent

Informed consent was not required for this research because this article does not contain any studies with human participants performed by any of the authors.

## Data availability statement

The datasets generated and analysed during the current research are available at the Zenodo: https://doi.org/10.5281/zenodo.8408356. Considering Twitter's terms and conditions, along with ethical and legal considerations, the dataset includes Tweet IDs to ensure the reproducibility of the research.

## Funding

This research was funded by the Internal Grant Agency of FEM CZU in Prague under Grant 2022B0009 — Application of artificial intelligence to regional segmentation using Big Data.

## CRediT authorship contribution statement

**Kateřina Kuralová:** Writing – review & editing, Writing – original draft, Visualization, Validation, Supervision, Methodology, Formal analysis. **Kristýna Zychová:** Writing – review & editing, Writing – original draft, Visualization, Validation, Methodology, Conceptualization. **Lucie Kvasničková Stanislavská:** Writing – review & editing, Funding acquisition, Formal analysis, Data curation. **Lucie Pilařová:** Writing – original draft. **Ladislav Pilař:** Writing – review & editing, Methodology, Data curation.

## Declaration of competing interest

The authors declare that they have no known competing financial interests or personal relationships that could have appeared to influence the work reported in this paper.
